# A Major Depressive Disorder in a Patient with Pseudobulbar Affect

**DOI:** 10.7759/cureus.3746

**Published:** 2018-12-18

**Authors:** Eduardo D Espiridion, Kyle N Risos

**Affiliations:** 1 Psychiatry, Frederick Memorial Hospital, Frederick, USA; 2 Osteopathic Medicine, Nova Southeastern University School of Osteopathic Medicine, Davie, USA

**Keywords:** pseudobulbar affect, major depression, suicide

## Abstract

Pseudobulbar affect is a condition that presents as involuntary laughing or crying among patients with certain neurological conditions or injuries. There is an outburst of crying or laughing that may not be connected to the current emotional state. Because pseudobulbar affect often involves crying, the condition is frequently mistaken for depression.

We present the case report of a 54-year-old male patient who had a stroke and who presented to his physician with a chief complaint of crying spells. His family expressed, and his physician believed, that he was suffering from depression because of his dramatic clinical presentation. The patient initially denied that he was depressed. Despite the denial, he was managed with psychotherapy and an antidepressant medication, Remeron (mirtazapine). The treatment did not improve his clinical symptoms. He was eventually treated with dextromethorphan/quinidine (DM/Q), 20 mg/10 mg, with a dramatic resolution of the crying spells.

However, psychosocial stressors, including the death of his father, job loss, and financial problems, made him depressed with vegetative symptoms. His crying spells came back and became more intense and frequent. He became worthless and hopeless. This depression was unrelated to his stroke. He was diagnosed with a major depressive disorder and was treated with antidepressants and psychotherapy. He experienced the depression several months after his crying spells resolved with the DM/Q. His recent bout of depression was treated with another antidepressant, vilazodone, and he was given a more intensive outpatient psychotherapy treatment. All the psychiatric symptoms, including the crying spells, have improved after treatment.

## Introduction

Pseudobulbar affect (PBA) has been reported with strokes and other neurological conditions, including amyotrophic lateral sclerosis, multiple sclerosis, traumatic brain injury, brain tumors, and Alzheimer's dementia. It presents as a frequent involuntary and uncontrollable outburst of crying or laughing. Because of the crying spells, PBA is frequently mistaken for depression. This condition is still under-recognized and under-treated. It is one of the lesser known symptoms of stroke. The tearfulness is not linked to the patient's mood because they may feel happy but will overtly cry and may not able to stop. There is a significant problem with their emotional expression rather than a subjective disturbance in their feelings. The patient's mood may appear normal between episodes. Subsequently, patients become anxious in public as they worry about future episodes. They feel confused, frustrated, and isolated with a reduction in their quality of life. 

## Case presentation

**Figure 1:**
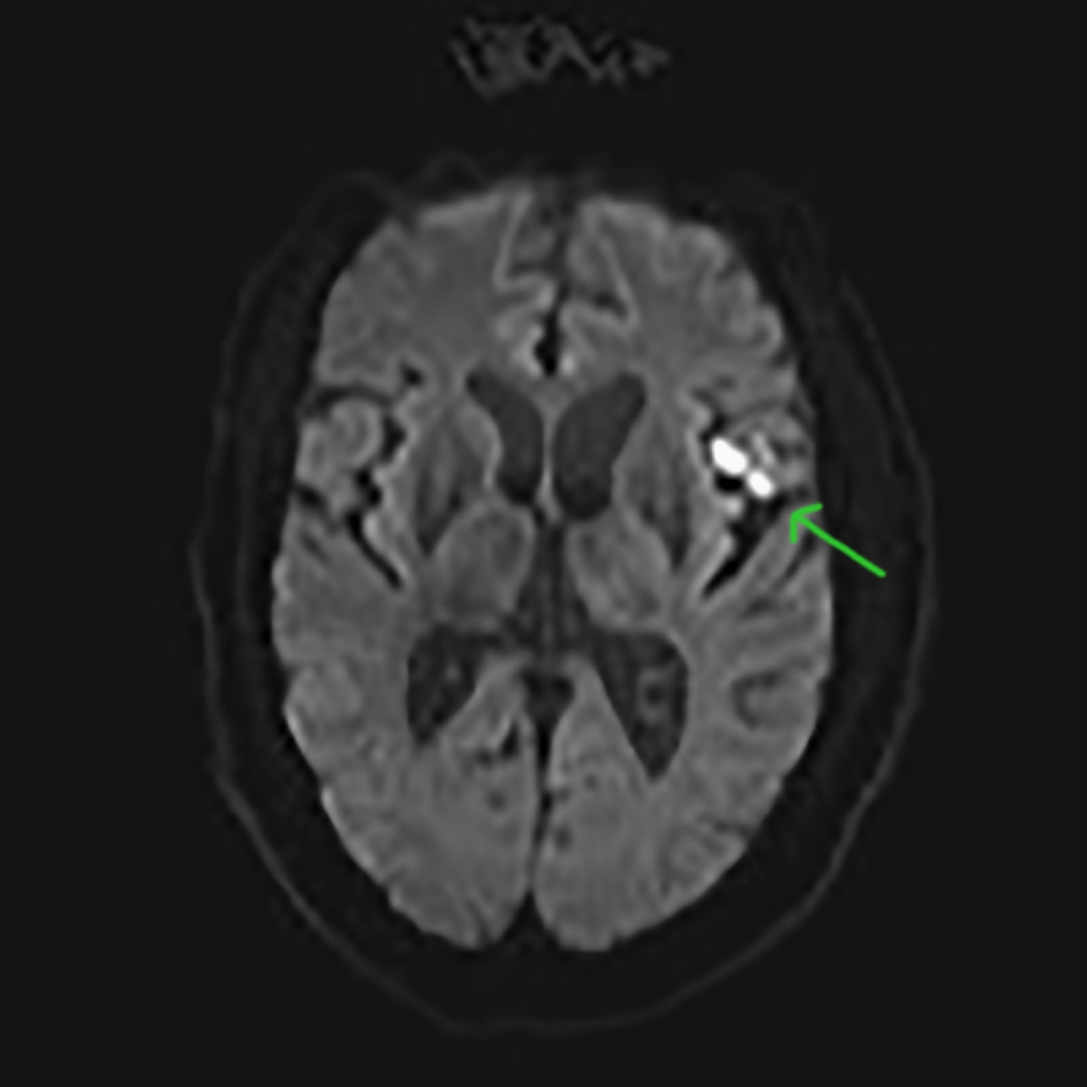
Magnetic Resonance Imaging (MRI) of the Brain Without Contrast MRI scan of the brain shows two small foci of increased signal intensity within the subcortical white matter of the left frontal lobe that represents subacute infarcts

This is a case of a 54-year-old divorced Caucasian male who has no prior psychiatric history or hospitalization. He was the lead singer of a local rock band. The patient has a 20-year history of cocaine abuse. He routinely uses cocaine prior to his stage performances. He reportedly binged on cocaine following a concert performance in a downtown bar. He snorted more cocaine than usual and immediately developed a severe headache and lower extremity numbness. He was rushed to the local emergency room, and an MRI of the brain without contrast was done. The MRI of the brain without contrast showed two small foci of increased signal intensity within the subcortical white matter of the left frontal lobe (Figure 1). 

**Figure 2:**
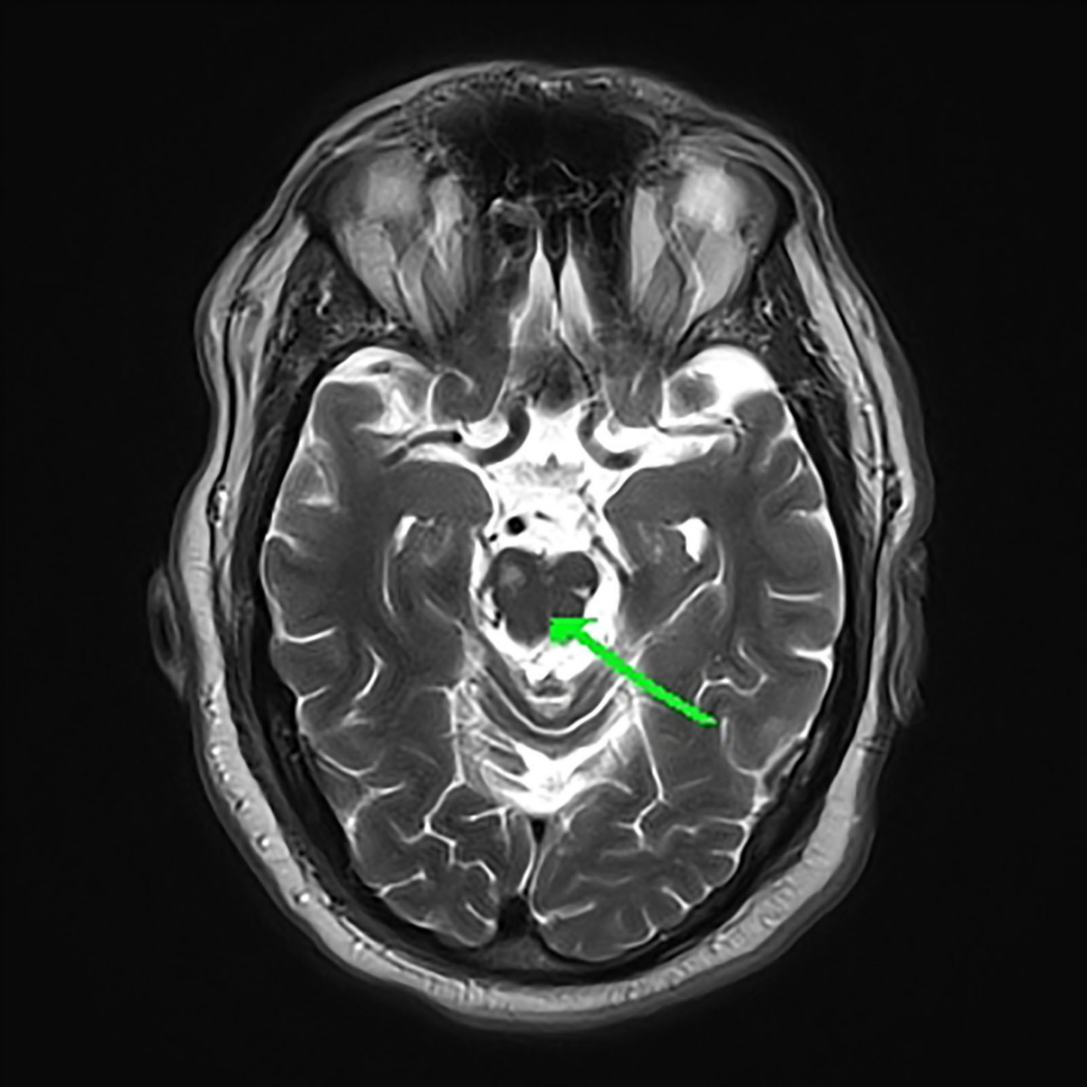
Magnetic Resonance Imaging (MRI) of the Brain Without Intravenous Contrast MRI of the brain shows a focus of subacute infarct involving the right side of the pons.

It also showed the finding of bilateral foci of infarct involving the right side of the pons (Figure 2). Both figures are consistent with an embolic phenomenon. 

The patient has not complained of any somatic issues related to his cocaine use. The patient reported some depressive and anxiety symptoms as a result of the stroke but he remained optimistic about his recovery. He denied any vegetative depressive symptoms or suicidal ideations. He was initially seen by a nurse practitioner at the emergency room who diagnosed him with an adjustment disorder. After stabilization in the medical floor and a two-week stay at a rehabilitation program, the patient was sent home with good family support. Over the course of two weeks, the patient noted significant crying spells most of the day, nearly every day. He consistently denied that he was depressed and suicidal. His family became concerned and sent him to his primary care physician. The physician believed that he was suffering from depression because of his dramatic clinical presentation. The patient was eventually referred to the local mental health center by his primary care physician because of uncontrollable crying spells. He was not subjectively depressed but objectively tearful with a flat affect. He also complained of sleeping difficulties with ruminative worries about his situation. He denied any suicidal thoughts. 

The patient was started on Remeron (mirtazapine), 15 mg at bedtime. He also engaged in weekly psychotherapy sessions. Over the next two months, the patient noted improvements in his sleeping patterns and appetite. The crying spells persisted. The patient was observed to be tearful while at the waiting area, during the psychiatric evaluation, and after his treatment appointment. His family reports that he cries every day for no apparent reason. Despite reassurances that he was not depressed, the family was convinced that his emotional state was getting worse. The patient was eventually diagnosed with pseudobulbar affect (PBA) because of his repeated outburst of involuntary crying. The crying was occurring even though there was no sad event that triggered those emotions. These episodes were persistent and had occurred in different situations or settings. He was referred to a local neurologist who confirmed the PBA. Eventually, he was managed with dextromethorphan hydrobromide and quinidine sulfate (DM/Q), 20 mg/10 mg capsules twice a day, in addition to his mirtazapine. The patient's crying spells improved significantly after the DM/Q was started. He tolerated it very well with no complaints of any side effects.

A year later, the patient had multiple tragedies in his family. His father, with whom he was very close with, suddenly and unexpectedly died. He also had an argument with his daughter, who later refused to talk to him. He was overwhelmed with financial problems. Because of these, the patient became more depressed and the crying spells recurred. Despite his medication compliance with DM/Q and mirtazapine, he noted worsening depression and occasional suicidal thoughts. He reported symptoms of sad mood, anhedonia, fatigue, excessive sleeping with early morning awakenings, increased appetite and weight gain, psychomotor retardation, and feelings of helplessness and worthlessness. He was having thoughts of shooting himself, even though he does not own a gun. This time, he was subjectively complaining of being "down in the dumps." He was seen in the emergency room for a crisis evaluation and referred back to the mental health center. He was reevaluated and his mirtazapine was switched to Viibryd (vilazodone) because of weight gain concerns. He also attended twice a week psychotherapy sessions. A month later, with these interventions, the patient's depressive symptoms, including the crying spells, had improved. He continues to receive his DM/Q and vilazodone and weekly psychotherapy sessions with no exacerbations of any mood symptoms.

## Discussion

Pseudobulbar affect (PBA) is a neuropsychiatric condition associated with various neurological conditions, including strokes. It remains under-recognized and often misdiagnosed with psychiatric conditions like depression and bipolar disorder. Early identification and prompt initiation of treatment are very important for these stroke patients. There was a narrative overview of PBA, including its epidemiology, pathophysiology, differential diagnosis, impact on public health, and therapeutic options. It remains a challenge to recognize and diagnose PBA around preexisting neurological conditions [1]. The PBA Registry Series trial was created to measure the prevalence of PBA among patients with strokes, Parkinson's disease, multiple sclerosis, amyotrophic lateral sclerosis, traumatic brain injury, and Alzheimer's dementia. Research involving the most current hypotheses (as to its physiopathology, clinical identification, and evidence for management) led to a treatment [2]. 

While its exact etiology is unknown, PBA likely results from disruptions in the brain structures and neurotransmitters that regulate emotions. A differential diagnosis of depression should be ruled out. Antidepressant therapies have been traditionally used but current treatment now involves combination agents employing multiple modalities [3]. Two tools are commonly used for screening of PBA: the Center for Neurologic Study-Lability Scale (CNS-LS) and the Pathologic Laughter and Crying Scale. The CNS-LS is a valid objective screening tool for PBA on patients. It is a seven-item questionnaire where each question has a range of score from one to five, with a maximum score of 35. The Pathologic Laughter and Crying Scales is an interviewer instrument comprising 18 questions with score ranges between zero and three for each question [4]. PBA is a common condition that affects approximately one in five stroke survivors at the acute and post-acute phases and one in eight stroke survivors beyond six months post-stroke. These prevalence rates are very important for clinicians [5]. 

Pseudobulbar affect is due to the dysregulation of three main neurotransmitter pathways, dopamine, serotonin, and glutamate, from the frontal cortical lobes through the cerebellum and brainstem, the corticolimbic-subcorticothalamic-pontocerebellar network. Stroke or infarct mediated interruptions of circuits projecting to the cerebellum and brainstem may result in disinhibition of well-controlled voluntary emotions, making them involuntary [6]. In our case presentation, the patient had a stroke involving the right side of the pons and the left frontal lobe. In another case report, the patient presented with crying spells and even suicidality, but she did not feel subjectively depressed. Her physicians felt that the underlying neurologic condition was due to a PBA and her symptoms were successfully treated with valproic acid [7]. Dextromethorphan-quinidine (DM/Q) has been shown to be effective to treat PBA. This can be managed well with this pharmacologic intervention. Dextromethorphan is an uncompetitive N-methyl-D-aspartate receptor antagonist, as well as a sigma-1 agonist. It affects several neurotransmitters in the brain, including both glutamate and serotonin. Serotonergic projections and receptors are widespread in the central nervous system. They are predominantly found in the brainstem and corticolimbic area and networks which may be involved in emotion. Glutamate is an excitatory neurotransmitter in the central nervous system. Quinidine is an optical stereoisomer of quinine. It is a pharmaceutical agent that acts as an anti-arrhythmic agent. DM/Q is a treatment in patients with stroke, dementia, or traumatic brain injury with PBA [8]. PBA can impact the quality of life as the disease burden is tremendous for the patient and it is independent with mood problems. Finding the right treatment impacts the symptoms that cause significant functional impairment [9]. The PRISM II Trial was an open-label, 12-week trial enrolling adults with PBA caused by dementia, stroke, or traumatic brain injury. All the study participants received DM/Q, 20/10 mg twice daily. Studies have evaluated fluoxetine, paroxetine, and citalopram trials in stroke patients with pseudobulbar affect with reduced severity and episode rates [10]. However, in the PRISM II trials, the dextromethorphan/quinidine medication showed improvement in the different efficacy scales that were used, including Neurologic Study-Lability Scale (CNS-LS) as primary efficacy measures, and the Clinical and Patient/Caregiver Global Impression of Change (CGI-C and PGI-C), Quality of Life-Visual Analog Scale (QOL-VAS), Patient Health Questionnaire (PHQ-9), and the Mini-Mental State Examination (MMSE). These are highly efficient screening and diagnostic tools that can help clinicians diagnose PBA in the community [11]. This patient case report points to the fact that a severe depression can occur in the setting of PBA. Both conditions have to be treated to improve their overall functioning and quality of life. 

## Conclusions

Strokes pose a significant burden on patients and their families. Pseudobulbar affect, which is a frequent presentation of strokes, requires early identification and prompt initiation of treatment. This condition can be managed with medication. However, clinical depression can happen in the presence of PBA, such as during the patient's reaction to the catastrophe of a stroke event, as well as significant changes in their everyday life. Major depression may occur as a result of these catastrophic events. Clinicians should have a presence of mind to assess for a mood disorder in strokes and treat them promptly as they affect that patient's quality of life. Dextromethorphan/quinidine is effective in controlling the symptoms of PBA, but antidepressant treatment and psychotherapy helped address the subsequent major depression episode.
